# Internal receptors in insect appendages project directly into a special brain neuropile

**DOI:** 10.1186/1742-9994-10-54

**Published:** 2013-09-10

**Authors:** Peter Bräunig, Katharina Krumpholz

**Affiliations:** 1 RWTH Aachen, Institut für Biologie II (Zoologie), Abteilung für Entwicklungsbiologie und Morphologie der Tiere, Helmertweg 3, Aachen, D-52074, Germany

**Keywords:** Insect, Locust, Thermoreception, Central projections, Leg, Mouthparts, Antenna

## Abstract

**Background:**

The great majority of afferent neurons of insect legs project into their segmental ganglion. Intersegmental projections are rare and are only formed by sense organs associated with the basal joints of the legs. Such intersegmental projections never ascend as far as the brain and they form extensive ramifications within thoracic ganglia. A few afferents of chordotonal organs of the subcoxal joints ascend as far as the suboesophageal ganglion.

**Results:**

We describe novel afferent neurons in distal segments of locust legs that project directly into the brain without forming ramifications in other ganglia. In the brain, the fibres terminate with characteristic terminals in a small neuropile previously named the superficial ventral inferior protocerebrum. The somata of these neurons are located in the tibiae and tarsi of all legs and they are located within branches of peripheral nerves, or closely associated with such branches. They are not associated with any accessory structures such as tendons or connective tissue strands as typical for insect internal mechanoreceptors such as chordotonal organs or stretch receptors. Morphologically they show great similarity to certain insect infrared receptors.

We could not observe projections into the superficial ventral inferior protocerebrum after staining mandibular or labial nerves, but we confirm previous studies that showed projections into the same brain neuropile after staining maxillary and antennal nerves, indicating that most likely similar neurons are present in these appendages also.

**Conclusion:**

Because of their location deep within the lumen of appendages the function of these neurons as infrared receptors is unlikely. Their projection pattern and other morphological features indicate that the neurons convey information about an internal physiological parameter directly into a special brain neuropile. We discuss their possible function as thermoreceptors.

## Introduction

The number, location and innervation of mechanoreceptors in insect legs have been studied intensively in the past, especially in locusts (for review see [1]). One conclusion from numerous studies from the 1980s [2-8] was that the axons of all mechanosensory neurons located in distal leg segments, that is all sensory fibres originating from neurons of trochanter, femur, tibia and tarsus, project only into their segmental ganglia. Only a few mechanoreceptive neurons of more proximal segments (coxa and subcoxa) may form intersegmental projections. All these older studies used heavy metal salts for staining, cobaltous chloride in most cases, and subsequent intensification with silver. Small-diameter axons, such as the ones from contact chemoreceptors, are very difficult to stain using this technique [9] perhaps also due to the toxicity of heavy metal salts. Less toxic staining agents such as Biocytin or Neurobiotin™ at that time were not yet available.

To exclude the possibility that sensory projections had been overlooked in previous studies, we started to reinvestigate this question by retrograde staining of a variety of nerves innervating leg sensory structures in migratory locusts using Neurobiotin™-staining. In the majority of cases no central projections could be revealed that had not already been described in previous publications [2-8]. A few stainings of leg nerve branches in the distal femur, however, showed one to three thin and weakly-labelled fibres that, in contrast to all others, ascended towards more anterior ganglia. In well-stained preparations it became obvious that these fibres terminate in a special protocerebral neuropile, previously named *superficial ventral inferior protocerebrum* (SVIP) [10,11]. These authors observed a few fibres terminating in this neuropile after staining maxillary and antennal nerves in locusts and crickets.

The objective of the present study was therefore to characterise these projections in more detail and to find out whether such projections originate from all appendages. Finally, we tried to identify the neurons in the legs that form these projections. In order to locate these neurons we stained circumoesophageal or cervical connectives retrogradely. Because of the long distances involved we used nymphs and sometimes even embryos to locate the cells. As will be shown here, the ascending axons belong to a few neurons with unusual morphology that are located within, or in close association with, peripheral nerves.

## Results

### Certain leg nerve branches contain fibres that project directly into the brain

**Figure 1:**
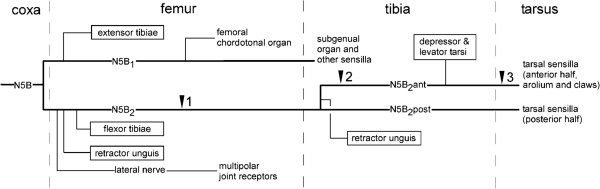
**Simplified diagram of the innervation of muscles and prominent sense organs of locust legs.** Please note that N5B_2_ in the proximal tibia splits into two major branches (N5B_2_ant, N5B_2_post) that proceed toward the tarsus. Triangles mark the sites where nerves were filled. Projections into the brain were only observed after filling the main nerve 5B_2_ in the femur (site 1) or its anterior tibial branch (sites 2 and 3). The numerous tegumentary branches of the major leg nerves (N5B_1_ and N5B_2_) that innervate the hair sensilla on the cuticle are not shown.

In locusts as in other insects, the great majority of muscles and sense organs in the legs are innervated by the main leg nerve, named nerve 5B of the thoracic ganglia (Nerve 5A is a much smaller branch in the thoracic cavity). Nerve 5B splits into two major branches, branches 5B_1_ and 5B_2_, in the distal coxa [12,13]. As outlined in Figure 1 nerve 5B_1_ innervates the extensor tibia muscle, sense organs in the femur and proximal tibia, and does not proceed further distal. Nerve 5B_2_ innervates all other muscles and sense organs of femur, tibia and tarsus. In the proximal tibia this nerve splits into two major branches that run along the ventral cuticle of the tibia, one anterior (N5B_2_ant) one posterior (N5B_2_post). Both branches proceed as far as the third (the most distal) tarsal segments, but arolium and claws only receive branches of the anterior nerve [13-15].

**Figure 2:**
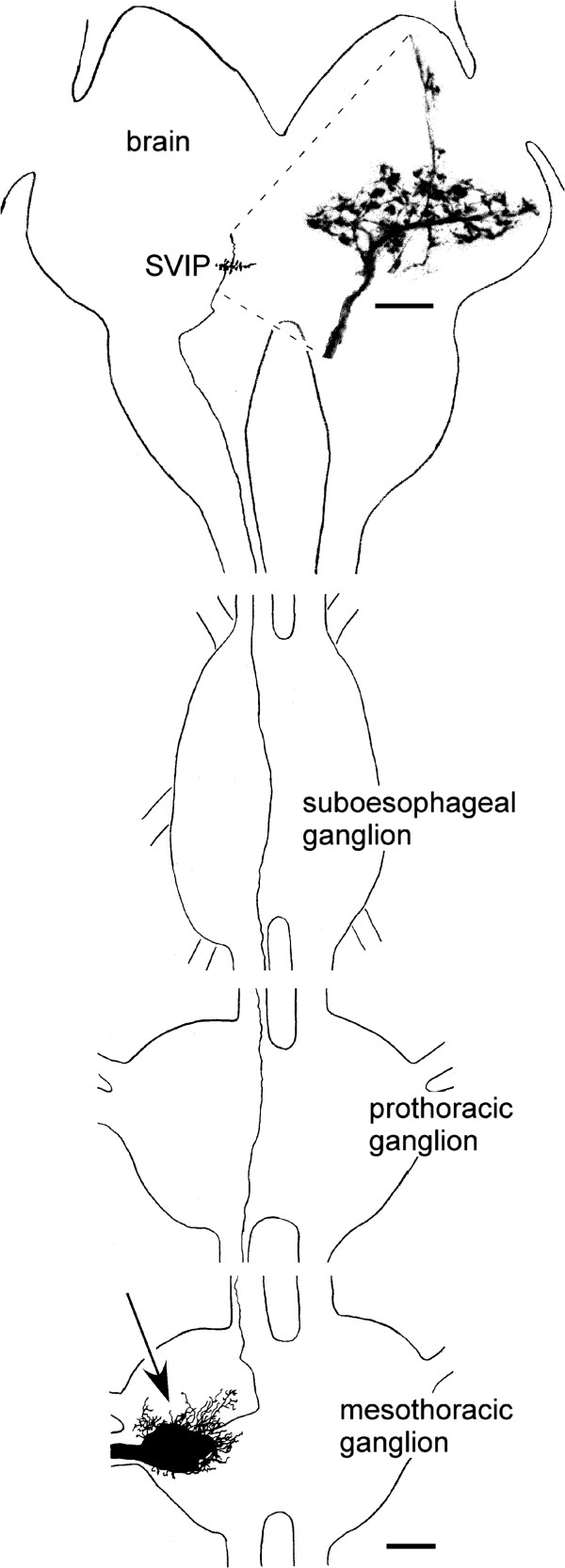
**Intersegmental projections from leg afferents.** Central projections as seen after filling the anterior branch of leg nerve 5B_2_ in the first tarsal segment of the middle leg in a third instar nymph (drawn from confocal data). Sensory fibres from tarsal sensilla form massive projections in the mesothoracic ganglion (arrow). In addition a single fibre ascends towards the brain without any side branches. The fibre terminates with numerous blebby endings in a neuropile called the superficial ventral inferior protocerebrum (SVIP). The inset shows a confocal image of these terminals at higher magnification and high gain. Scale: 100 μm, 20 μm in inset.

Sensory projections ascending from thoracic ganglia towards the brain were first observed after staining nerve 5B_2_ in the distal femur (site 1 in Figure 1). Stainings of nerve 5B_1_ or the lateral nerve [16] at about the same level did not show such fibres. More selective stainings of nerves in tibia (site 2 in Figure 1) and tarsus (site 3 in Figure 1) showed that such projections are only observed when staining the anterior branch of nerve 5B_2_ in the tibia or the first tarsal segment. When staining this nerve in the third tarsal segment such projections were no longer observed. Complete staining of the ascending fibres in adult locusts failed. Even when using smaller nymphal stages complete staining was difficult to achieve. Figure 2 shows one of the few examples for a successful complete staining of one such fibre obtained after staining nerve N5B_2_ant in the first tarsal segment of a mesothoracic leg in a third instar nymph. While all other sensory fibres form only local ramifications within the ipsilateral half of the mesothoracic ganglion this fibre proceeds without any ramifications as far as the brain. Only here the fibre forms ramifications with varicose terminals (Figure 2, inset). When staining nerve N5B_2_ant in the distal tibia a maximum of two such fibres were observed, when staining nerve 5B_2_ in the femur a maximum of three.

### Ramifications within the brain

**Figure 3:**
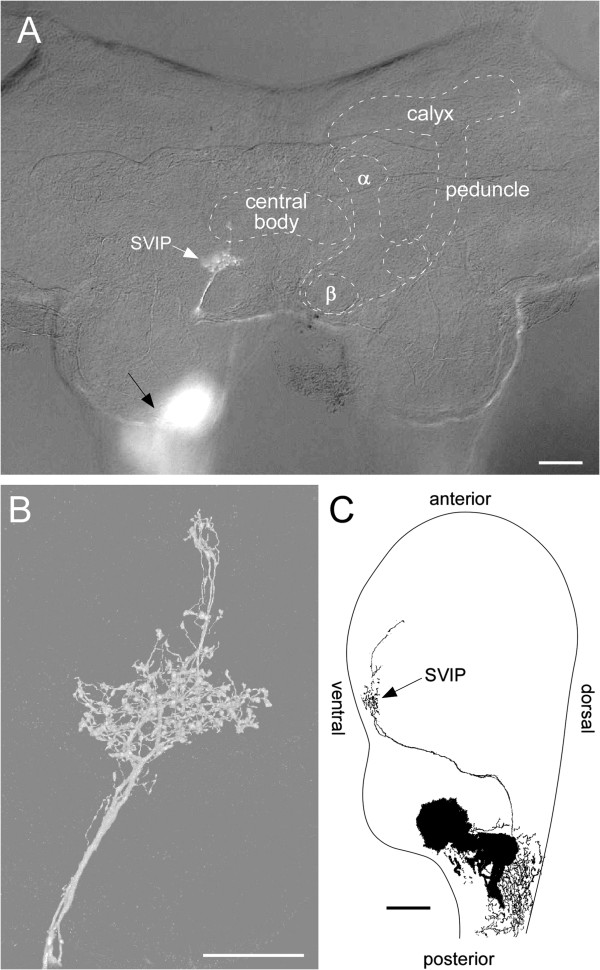
**Projections in relation to brain anatomy. A** Anterior view of the brain of a 4th instar nymph after staining the maxillary palp nerve as seen in a double exposure using differential interference contrast and epifluorescent illumination. The principal neuropiles of the brain are outlined. The black arrow points out projections in deuto- and tritocerebrum, the white arrow points at ramifications within the superficial ventral inferior protocerebrum (SVIP). **B** The ramifications within the same preparation as seen in the confocal laser-scanning microscope and at higher magnification. **C** Reconstruction of the projections into the SVIP based on 6 consecutive 30 μm sagittal sections of a preparation similar to the one shown in **A** and **B**. Please note the extreme ventral position of the projections. Scales: 100 μm in **A**, **C**; 50 μm in **B**.

**Figure 4:**
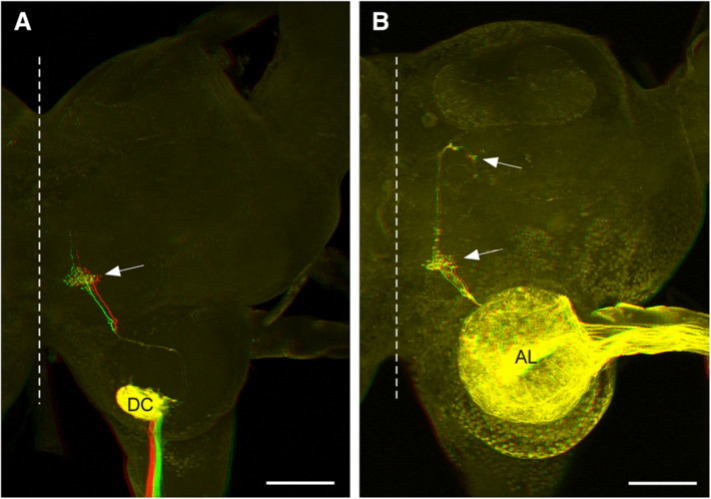
**Projections from maxillary and antennal nerves.** Fibres projecting into the superficial ventral inferior protocerebrum (SVIP) as seen after filling nerves of head appendages (anaglyphs for viewing with red/green glasses; midline of brain indicated by broken line). **A** Projections into the SVIP (arrow) after staining the maxillary palp nerve. Massive projections of palpus chemosensory afferents in the deutocerebrum (DC) are also stained (those in the tritocerebrum are out of the focal plane). **B** Projections after filling one of the two major branches of the antennal nerve in the distal flagellum. Apart from projections in the antennal lobe (AL) of the deutocerebrum a few fibres project into the protocerebral SVIP (arrows). Scales: 200 μm.

In the brain most ramifications of these leg afferents are restricted to a small protocerebral neuropile region. This neuropile region had already been shown to receive similar projections from the maxillary palps and antennae in crickets and locusts by Ignell et al. [10,11] and was named the “superficial ventral inferior protocerebrum” (SVIP). Because the leg afferents were difficult to stain due to the distances involved, we stained the maxillary palp nerves in order to get more intense labelling (Figures 3, 4A). The ramifications with their characteristic varicose terminals were much more distinct than those from stained from the legs (Figure 3B). Within the suboesophageal ganglion, the tritocerebrum, and posterior deutocerebrum the fibres are completely obscured by numerous other sensory fibres (Figure 3A), but there are clearly only 2–3 fibres that proceed as far as the protocerebrum in such preparations. With differential interference contrast it is possible to discern the principal neuropiles such as mushroom bodies and the ellipsoid body even in wholemounts of the brain, at least in younger nymphal stages. Combining this with epifluorescent illumination showed the SVIP close to the β-lobe of the mushroom body, but slightly more anterior and medial (Figure 3A). With respect to the neuraxis of the CNS the SVIP is located in the extreme ventral region of the protocerebrum (Figure 3C).

Similar projections were observed after staining antennal nerves. In addition to numerous fibres terminating within the olfactory lobes of the deutocerebrum a few fibres clearly project into the SVIP (Figure 4B). The main antennal nerve splits into two major branches within the scapus. Both branches proceed towards the distal tip of the flagellum. Fibres projecting into the SVIP were observed after staining either one of these major branches. Two to three fibres appeared after staining these nerves from either the pedicellar region or from a distal region, 4–5 annuli proximal to the very tip of the flagellum, indicating that the neurons of origin are located close to the tip of the antenna.

### Staining connectives labels neurons in tibiae and tarsi

**Figure 5:**
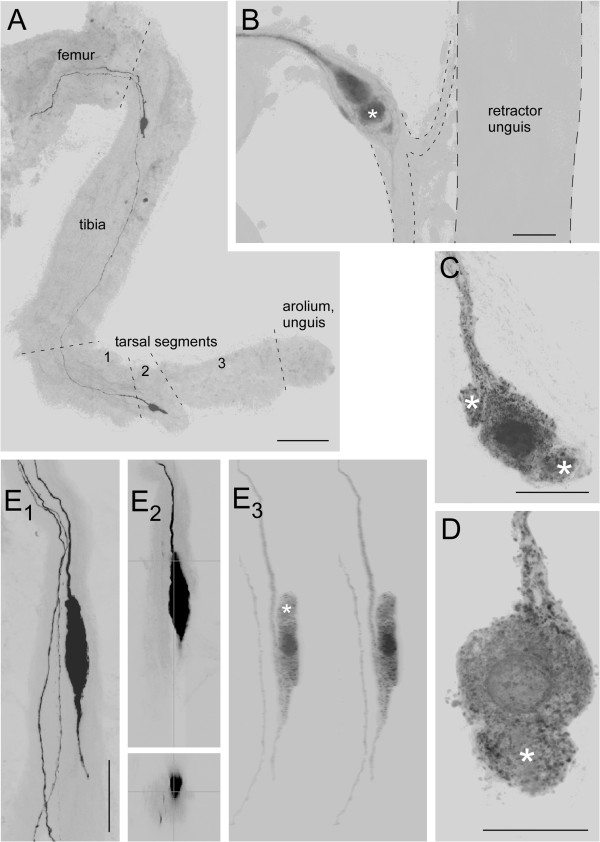
**Neurons in the legs of embryos and nymphs as seen after filling circumoesophageal or cervical connectives. A** The middle leg of an embryo at 70% of development (borders between leg segments indicated by stippled lines). Typically one neuron is located in the proximal tibia, a second neuron in the second tarsal segment. **B** A neuron in the middle leg of a fourth instar nymph located in the motor nerve (outlined by stippled line) of the proximal tibial part of the retractor unguis muscle (outlined by broken line). **C** and **D** Further examples for such proximal tibial neurons at higher magnification. Please note the bulge-like extensions of the neurons (white asterisks). **E** Another example where the neuron is located within the major nerve 5B_2_. E1 shows this neuron and two additional fibres proceeding further distal. E2 provides z- and y-projection of the same confocal stack illustrating the location of the soma within the nerve. In the high-gain projection (E1) the neuron looks as if there is a long distal process. E3 provides a 3D stereo pair obtained at lower gain to show that this distal “process” is formed by the axon that first bypasses the soma, makes a U-turn and returns to the soma. Also in this neuron that looks more elongated than the neurons shown in **B-C** a bulge-like extension of the soma is discernable (white asterisk). Distal is down in all panels. Scales: 100 μm in **A**, 20 μm in **B-D**, 40 μm in **E**.

In order to elucidate the origin of the fibres, either the cervical or the circumoesophageal connectives were filled in nymphs and embryos. Such stainings labeled neurons in the legs that differed from all known insect sensory neurons. In nymphs (n=5, 15 legs inspected) the success rate was very low even with incubation times of several days. In successful preparations usually only a single neuron in the proximal tibial region was labelled, and only in the fore and sometimes the middle legs. The neurons had a variable position and appeared to be associated with the one part of the retractor unguis muscle that inserts in the proximal tibial region (Figure 5B). Where exactly with respect to this muscle the neurons were located remained unclear because under epifluorescent illumination internal structures within the legs were hard to discern. In most cases, however, the neurons looked as if they were located within peripheral nerves in the vicinity of this retractor unguis. In addition to the axon belonging to this neuron, often one or two other stained fibres could be seen that proceeded into more distal regions of the tibia where staining faded (Figure 5E).

Staining connectives in embryos (n=12) yielded more consistent results (Figure 5A). Here neurons were stained in all legs most likely because of the reduced distances. Again there was one neuron in the proximal tibial region. This neuron failed to stain in only 4 of all legs examined (n=72). In addition, one or two neurons showed up in the tarsal segments. In about 50% of all legs examined a neuron was located in the ventral region of the second tarsal segment. In the other 50% an additional neuron was stained. This second neuron was either located close to the first one, or in variable positions in the third tarsal segment.

The morphology of the neurons observed in embryonic legs differed from that of the neurons in nymphs. In the embryos the neurons had what appeared to be a rather short and stout neurite (Figure 5A). In the nymphs the neurons appeared to have one or two amorphous bulge-like protrusions (asterisks in Figure 5B-E) that neither resembled the numerous slender ramifying dendrites usually observed with multipolar insect sensory neurons, nor with the rod-like dendrites of bipolar sensory neurons.

### Neurons in the proximal tibia

**Figure 6:**
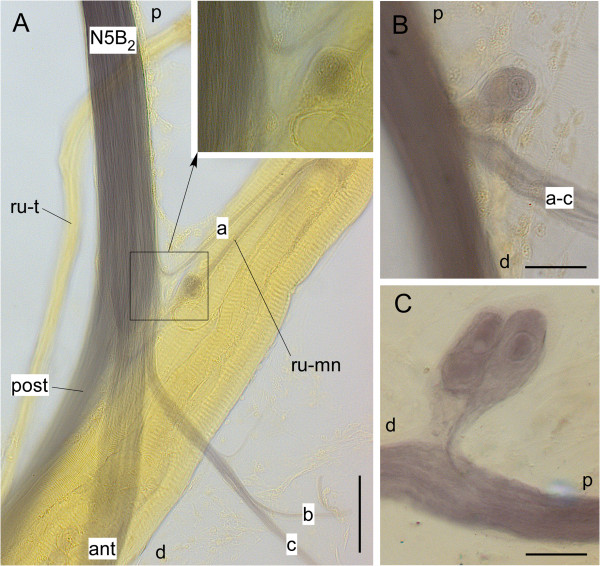
**Tibial neurons as seen after staining nerves with nickel chloride. A** Proximal tibial region of a hind leg showing the split of nerve 5B_2_ (N5B_2_) into its anterior (ant) and posterior (post) branches, the sensory branches (a-c), and the motor nerve to the retractor unguis muscle (ru-mn). In this example the neuron is located within the motor nerve (see inset). **B** and **C** show two other examples where the neuron is associated with the common root of nerves a-c **B** or connected to the main nerve (N5B_2_) by a short connection **C**. d=distal, p=proximal, ru-t=tendon of retractor unguis. Scales: 50 μm in **A**, 20 μm in **B** and **C**.

Staining the connectives in embryos might have revealed neurons that were not yet fully differentiated. Staining in nymphs had a low rate of success, did not reveal the exact position of the neurons in relation to other internal structures, and the long incubation times might have caused artefacts. For these reasons in older nymphs and adult locusts leg nerve branches were filled with nickel chloride to stain the cells with shorter incubation periods and over shorter distances. Additional advantages are that in such preparations usually all other nerve branches are stained and that they can be examined with transmitted light that makes the visualisation of other tissues as well as further dissection easier than with epifluorescent illumination. Figure 6 shows examples of such stainings of the tibial neuron in hind legs. Here nerve 5B_2_ was stained in the distal femur (n=12) which usually resulted in complete staining of all major nerve branches in the proximal tibia. As mentioned above, this nerve splits into two major branches that proceed into the tibia; an anterior branch and a slightly thinner posterior branch. The anterior branch passes around the anterior face of the retractor unguis bundle, the posterior branch passes behind this muscle. Close to the split between these major branches the anterior nerve gives off several smaller branches. One branch innervates the retractor unguis muscle (ru-mn in Figure 6A), three other small branches (a-c in Figure 6A) are sensory and innervate hair sensilla and campaniform sensilla on the posterior face of the proximal tibial region (those of the anterior face are innervated by branches of nerve 5B_1_[7]). The sensory nerves may emerge separately from the anterior branch of nerve 5B_2_ or they may have a common root. In four of the preparations a cell could be observed associated with one of these smaller nerves. It was located within one of the sensory branches or within the retractor unguis motor nerve (Figure 6A,B), or it was attached to the main nerve 5B_2_ by a very short connection (Figure 6C). As already observed after Neurobiotin™ staining, unusual globular protrusions were associated with the cells. An extreme example is shown in Figure 6C. Here the large cell to the right appears to be associated with two smaller ones. All three are enveloped by a kind of transparent capsule. When focusing through the entire structure, however, these putative smaller cells lack a nucleus as can be clearly seen in the large cell. Also there is clearly only one axon entering the main nerve.

### Cells in the tarsal segments

**Figure 7:**
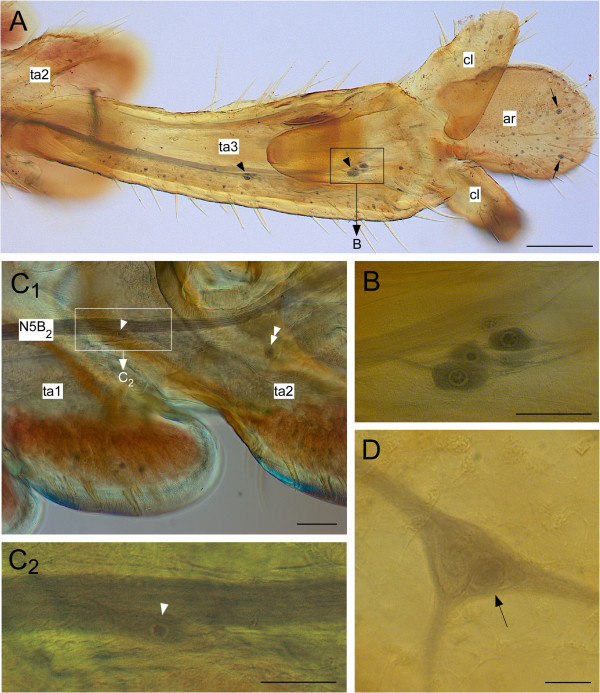
**Tarsal neurons as seen after staining nerves with nickel chloride. A** shows tarsal segments 2 and 3 (ta2, ta3), arolium (ar), and claws (cl). The numerous blue dots are the somata of sensory neurons. Most prominent are the somata of sensory neurons in two scolopidial organs (arrowheads) and that of two large campaniform sensilla on the arolium (arrows). **B** The neurons of the distal scolopidial organ at higher magnification. Please note the typical bipolar morphology of these cells in comparison to the cells shown in **C** and **D**. **C**_**1**_ The root of a side branch of the anterior nerve 5B_2_ (N5B_2_) contains a soma. **C**_**2**_ The same neuron at higher magnification. **D** Another example for a soma within a nerve branch point. Again a bulge-like extension of the neuron is visible (arrow). Scales: 200 μm in **A**, 50 μm in **B** and **C**_**2**_, 100 μm in **C**_1_, 20 μm in **D**.

After staining tarsal nerves (n=10) similar cells could be observed. Figure 7A shows an example for staining the anterior nerve 5B_2_ from the first tarsal segment. In such stainings almost all sensory cells associated with cuticular or internal sensilla are labelled, including clusters of small bipolar neurons associated with contact chemoreceptors. In addition to these numerous sensory cells one (n=6) or two (n=3) cells appeared to be associated with nerve branches that innervate the anterior pulvillus of the second tarsal segment (Figure 7C,D). Only in one preparation a cell was labelled in the third tarsal segment. All these cells were clearly not associated with any tegumentary sensillum and lacked the typical bipolar morphology of sensory cells associated with sensory hairs, campaniform sensilla, and scolopidial cells (Figure 7B). One such cell was further exposed by cutting away unstained tissue that usually impairs visibility in wholemount preparations. This tarsal cell also showed the additional bulge-like structure described above for the tibial cells (Figure 7D).

### Ultrastructure

**Figure 8:**
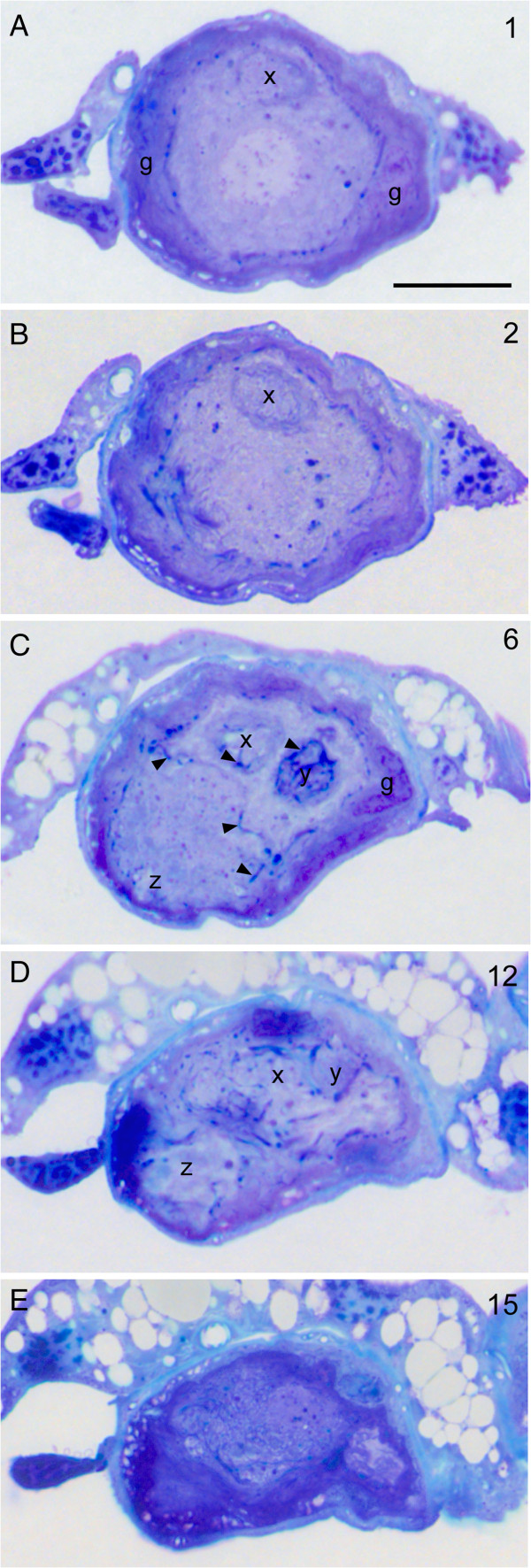
**The tibial neuron in semi-thin sections. A**-**E** show examples from a series of consecutive semi-thin sections starting at the soma **A** close to the retractor unguis motor nerve and proceeding further distal (the arabic numerals on the right give the section number). The soma is surrounded by a thick glial capsule (g, dark blue staining) that decreases in thickness in distal regions **D, E**. Distally the cytoplasm is invaded by glial tendrils (arrowheads) and contains areas that in the electron microscope look like bundles of small dendrites surrounded by glial processes (x, y, z; compare Figure 9) Scale: 20 μm.

**Figure 9:**
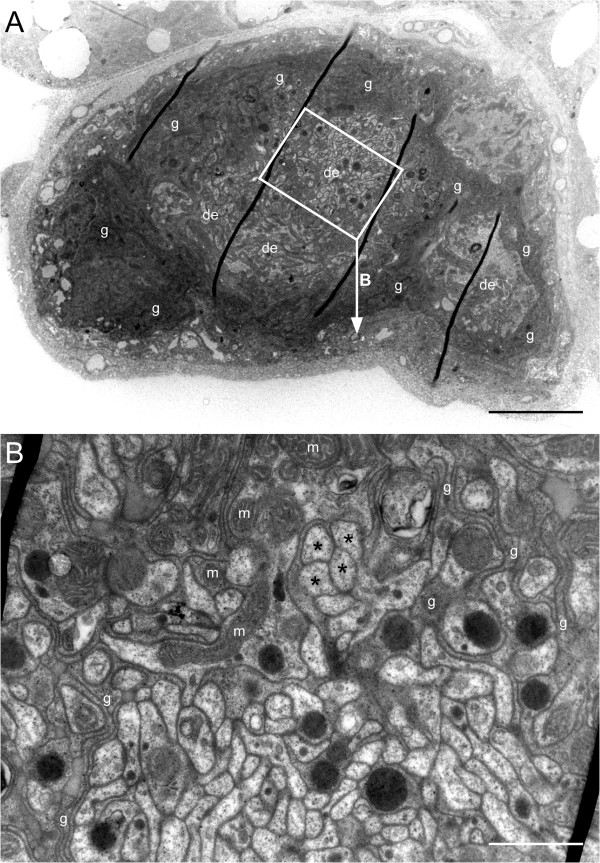
**Ultrastructure of the tibial neuron. A** shows a low-power transmission electron microscopic photo through the area with dendritic ramifications (de) and their glial sheath (g) which corresponds to the semi-thin section shown in Figure 8E. The oblique dark lines are folds in the section. The rectangle indicates the area shown in **B**. **B** shows an area filled with dendritic tangles. It is characterised by numerous small profiles (a few marked by asterisks) surrounded by glial structures (g). One of the larger dendritic profiles contains many mitochondria (m). Scales: 5 μm in **A**, 1 μm in **B**.

As mentioned above the appearance of the cells in the light microscope was hard to interpret. The next logical step, the examination of the cells within the transmission electron microscope at first seemed impossible for two reasons. First, it is almost impossible to locate the cells within fresh, unstained specimens. Second, their position varies considerably. In very rare cases, however, the cell is attached to the main nerve by a short stalk (Figure 6C). Because such a structure should be visible also in fresh specimens (provided it survives dissection) we dissected legs in the proximal tibial region. In about one out of thirty legs opened, the cell could thus be located. We managed to successfully dissect four such cells for embedding. During inspection of series of semi-thin sections (Figure 8) it became clear that the bulge-like extensions of the cells (Figures 5, 6) that in nickel-stained specimens may look like additional cells (Figure 6C) are parts of a single cell. In consecutive sections they developed within the cytoplasm as roughly circular regions enveloped by strongly counterstained material. In ultrathin sections these intensely counterstained regions were discernable as glial filaments that invade the cell and surround these circular areas. The glial protrusions enveloped tangles of small-diameter profiles that we interpreted as dendritic ramifications. Larger dendritic ramifications, intermingled with the small ones, contain many mitochondria (Figure 9).

## Discussion

### New afferents in locust appendages

The major result of the present study is the demonstration that there are direct sensory projections from the appendages into the protocerebrum in locusts. These projections originate from a new type of neuron that appears to be located within or closely associated with peripheral nerve branches in defined regions. The axons of the neurons project into a defined protocerebral neuropile area located ventrally in the brain (SVIP). Here they form characteristic varicose terminals. While passing through other ganglia of the ventral cord these axons do not form any side branches.

Afferent projections in this particular protocerebral neuropile were already observed after staining maxillary nerves in locusts and crickets [10]. These authors already noted its extreme ventral location (with respect to the neuraxis) in the vicinity of the tips of the beta-lobes of the mushroom bodies. Most terminals observed here and in previous studies [10,11] terminated within the SVIP but a few extended further anterior into undefined protocerebral territory. These additional projections are most prominent after staining the antennal nerves (Figure 4B). Similar projections were also observed after retrograde labeling of antennal nerves in the cockroach, *Periplaneta americana*[17], and the stick insect, *Carausius morosus* (Jens Goldammer, personal communication).

The exact location of the neurons in antenna and maxillae remains unknown for the following reasons. Because of their intersegmental projections the locations of the somata in the legs could be retrogradely labeled by staining connectives. Since there are no intersegmental projections of flagellar neurons this strategy fails in this case. It also fails with the neurons located within the maxillary palps. Hundreds of maxillary afferent neurons project intersegmentally through the circumoesophageal connectives and terminate in glomerular regions of the trito- and deutocerebrum ([10,18]; Figure 4A). Staining the circumoesophageal connectives would in turn stain hundreds of sensory neurons in the maxillary palps and it would be impossible to identify the few neurons that project into the SVIP. Indirectly, however, our results indicate that such neurons are located within very distal regions of the antennal flagellum. In the maxilla they only occur in the palpus.

Thus neurons with projections into the SVIP are located in the telopodites of the appendages (tibia and tarsus of the legs, maxillary palps, antennal flagellum). Such neurons appear to be absent from mandible and labium. The insect mandible is regarded as gnathobasic appendage that, during evolution, lost its telopodite [19-21]. Within this context the absence of such neurons in the mandibular nerve seems plausible. This interpretation does not apply to the labium. Like the maxillae the labium has telopodites in form of labial palps. Staining the labial nerve (nerve 5 of the suboesophageal ganglion), however, in no case revealed the typical projections into the SVIP. This indicates that nerve cells corresponding to the ones located in the legs, antennae and maxillary palps are either absent from the labial palps for an unknown reason or do not project intersegmentally into the brain.

**Figure 10:**
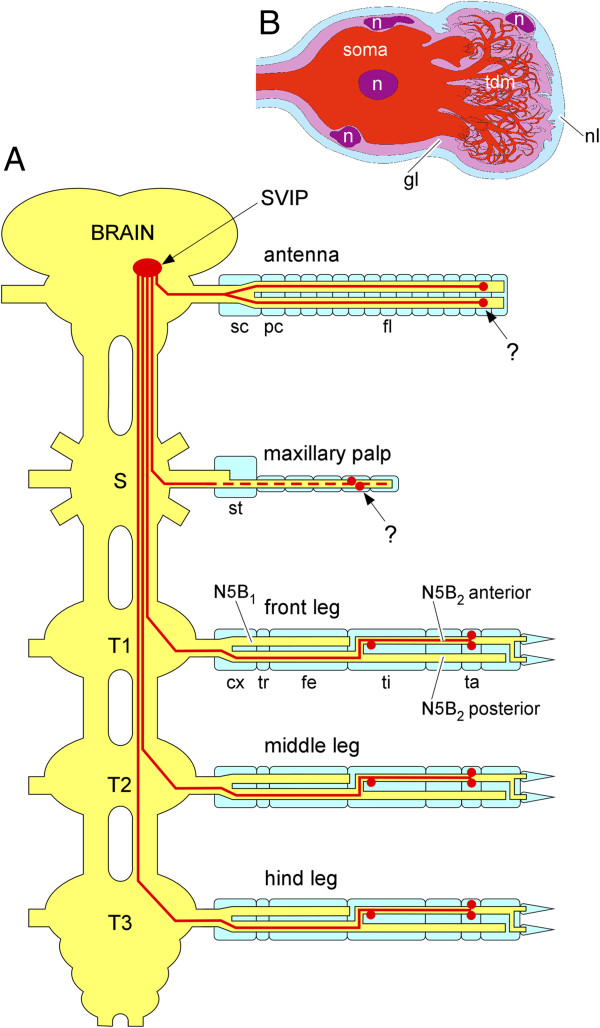
**Summary diagrams of the results of the present paper. A** All appendages except for the labial palps and the mandibles contain neurons that project into the superficial ventral inferior lateral protocerebrum (SVIP). Within the legs usually one neuron is located in the proximal tibia, one or two in tarsal segments. The exact location of the neurons within the maxillary palp is not known (question mark). Within the antenna each of the two major branches of the antennal nerve contains neurons in distal regions. Their exact position is not known (question mark). cx, coxa; fe, femur; fl, flagellum; pc, pedicellus; S= suboesophageal ganglion, sc, scapus; st, stipes; T1-T3, thoracic ganglia; ta, tarsus; ti, tibia; tr, trochanter **B** Schematic view of the structure of the neurons. Both, the soma and the terminal dendritic mass (tdm) are surrounded by a glial sheath (gl) and the neurilemma (nl). n, nuclei.

All these results taken together indicate that most appendages contain a few unusual afferent neurons that project into a special neuropile of the brain. A summary diagram is provided in Figure 10A. Their axons are very thin which indicates that there is no need to convey their information rapidly. This, and the location of the somata within or close to peripheral nerves, makes it likely that the neurons respond to a very special but as yet unidentified internal physiological parameter that varies relatively slowly over time. The information from all appendages is conveyed to the protocerebrum exclusively.

### Functional aspects

The morphology of the neurons described here in both the light and the electron microscope was clearly different from that of other insect sensory neurons found in the periphery. First they were not associated with any discernable accessory structures. Second, they did not resemble the typical bipolar sensory neurons found in chordotonal organs, sensory hairs, and campaniform sensilla [22-24]. Their morphology also differed from multipolar neurons associated with leg proprioceptors [5,25-28]. Finally the neurons were most often located within peripheral nerves.

After Neurobiotin™ -staining the neurons typically showed one or more bulge-like extensions the true nature of which could not be discerned (Figure 5). In fact in the beginning we interpreted these structures as an indication that the neurons had deteriorated during the long incubation times needed to stain them. The ultrastructural investigation, however, suggested that these bulge-like structures correspond to the tangles of small dendritic ramifications as revealed by the electron microscope (Figure 9). Nevertheless we cannot rule out that these tangles of delicate dendritic ramifications were damaged to some extent during retrograde staining or subsequent processing.

The ultrastructure of the neurons described here to a large extent resembles that of infrared receptors of the Australian fire beetle *Merimna atrata*. These infrared receptors are multipolar neurons that also form amorphous tangles of thin dendritic ramifications rich in mitochondria and sheathed in glial processes. Such tangles of small-diameter dendritic ramifications were named “terminal dendritic mass” [29,30]. Figure 10B provides a diagrammatic summary of the morphology of the type of locust neuron described here and its similar terminal dendritic mass.

Although it has been shown that locusts are able to perceive infrared radiation [31], there is one significant difference between the beetle infrared receptors and the neurons described here. In the beetle the neurons are closely associated with a specialized region of the cuticle so that warming of the cuticle can be immediately detected. The neurons described here are located far from the cuticle within peripheral nerves deep in the lumen of the appendages. This location precludes a function as infrared receptors. Radiant heat would be dissipated in the cuticle, the haemolymph and other overlying tissues.

The similarity to the beetle infrared receptors tempted us to speculate that the locust neurons could perhaps be a special kind of thermoreceptor. All insect thermoreceptors described so far are located on the cuticle, most of them on the antennae (for review see [32,33]). Such receptors might be suitable to measure the temperature of the surrounding environment. Like many other animals, locusts are known to perform behavioural thermoregulation [34,35]. For this behaviour the ability to measure the internal temperature, within the body and/or within appendages, might be much more relevant than measuring the environmental temperature. First electrophysiological experiments indicate that the neurons in the locust tibia might respond to cooling. This is corroborated by older observations that indicate the presence of cold receptors located in the tarsal segments of cockroaches [36]. This, however, does not rule out that the sensory neurons described here measure some other internal physiological parameter. For electrophysiological investigations, however, the same extremely difficult dissection would have to be used as for the ultrastructural investigation. Thus the functional investigation of these internal receptors is not going to be an easy task.

## Conclusions

We have identified a novel type of afferent neuron located within almost all locust appendages. These neurons send their axons directly into a special neuropile of the protocerebrum. Previous investigations in other insects show similar projections and thus indicate that similar neurons may be present in insect appendages in general. Morphologically these neurons differ from the great majority of peripheral sensory neurons so far identified in insects. They bear no resemblance to the typical bipolar neurons associated with tegumentary mechano- and chemoreceptors, nor with multipolar neurons found in stretch receptors or muscle receptor organs. In contrast to the latter, they are not associated with any accessory cuticular structures or tendons. They show resemblance to certain insect infrared receptors, but in contrast to these they are not associated with the cuticle but are found deep within the lumen of the appendages. We conclude that they represent a new type of afferent neuron that measures an internal physiological parameter, perhaps internal temperature, and conveys this information directly to the brain. As such they may be involved in behavioural thermoregulation.

## Materials and methods

### Insects and dissection

Adult locusts, *Locusta migratoria* or *Schistocerca gregaria*, as well as *Locusta* nymphs (3.-5. nymphal stages) and embryos were obtained from our own crowded cultures. The insects were anaesthetised by cooling to 6°C throughout dissection. For staining leg nerves the insects were restrained with pins on a piece of Balsa wood. Care was taken that the pins did not cause any injuries. Nerves were exposed by opening up the legs in appropriate locations (see Figure 1) and subsequent removal of overlying tracheal and connective tissue. After staining, the chain of ganglia from the brain to the fourth abdominal ganglion was dissected.

For staining antennal and mouthpart nerves isolated heads were used. The antennal nerves were exposed in the proximal region by opening scapus and pedicellus (n=8). Nerves in distal flagellar regions were exposed by gripping the fifth annulus from the tip with forceps and pulling the distal annuli away (n=10). For staining mouthpart nerves the suboesophageal ganglion was exposed by removing hypopharynx and bending the mandibles laterally (for details see [37]). The nerves entering the mandible as well those proceeding into the maxillary or labial palps were stained at least five times each.

For staining circumoesophageal or cervical connectives the dorsal part of head and thorax as well as the posterior half of the abdomen were removed. The ventral half was pinned into a Sylgard™-lined dish and covered with locust saline [38]. Removal of the tentorium, the salivary gland, some muscles and fatty tissue exposed the ventral nerve cord. The large tracheal trunks supplying the thoracic ganglia were carefully dissected in the region behind the third thoracic ganglion and opened up to the air at the saline surface. Finally the connectives were exposed and cut for staining.

For staining *Locusta* embryos, egg pods were collected immediately after deposition and incubated at 34°C on moistened cotton pads in Petri dishes. At this temperature *Locusta* embryos develop within 10 days after fertilisation, so 1 day roughly corresponds to 10% development. Exact staging of embryos followed Bentley et al. [39]. Embryos at 60%, 70%, or 80% development were removed from the eggs and opened by an incision along the dorsal midline. Removal of the yolk-filled gut exposed the CNS. Because older embryonic stages already have a cuticle that impedes penetration of chemicals, during fixation legs were perforated with small insect pins in dorsal regions of femur and tibia (where no major nerves run) to provide access for histological media. For the same purpose the legs of nymphs were opened by dorsal longitudinal incisions before fixation. Only for staining tarsal nerves with nickel chloride adult *Schistocerca gregaria* were used in addition to *Locusta migratoria*. In *Schistocerca* the tarsal cuticle is not as darkly pigmented as in *Locusta* which aids visibility of internal structures. All figures show data obtained using *Locusta* except for Figure 7.

### Staining procedures

The stump of a cut nerve or connective was isolated in a small vessel formed of Vaseline™ and briefly exposed to distilled water. Incubation was in Neurobiotin™ (5% (w/v) in distilled water, Vector Laboratories) at 6°C for 2–3 days when staining nymphs or adults, overnight when using embryos. After fixation of tissues in formaldehyde (4% in distilled water) for at least 2 h, preparations were washed for at least 2 h in several changes of phosphate buffered saline containing 0.1% Triton X 100 (PBS-TX). Subsequently tissues were incubated in CY3™-conjugated streptavidin (1:2000 in PBS-TX; Jackson Immuno Research) for 24–36 h. After washing in several changes of PBS-TX for 3 h, they were dehydrated in a graded series of isopropanol and cleared in methyl salicylate. Two brains of adult locusts with projections stained from the maxillary nerve were rehydrated, embedded in gelatine and cut into 30 μm vibratome sections. Sections were mounted on subbed slides, dehydrated and cleared as described above.

To study branching patterns of leg nerves, these were stained with 2% (w/v) nickel chloride overnight at 6°C or 2–3 h at room temperature. After removing the Vaseline well, the preparation was covered with saline and nickel was precipitated by adding 1 drop rubeanic acid solution (saturated solution in 100% ethanol) per ml saline [40,41]. After a brief wash in saline tissues were fixed either in formaldehyde (4% in distilled water) or alcoholic Bouin’s fixative for at least 2 h. They were dehydrated and cleared as described above.

### Light microscopy

A compound microscope (Zeiss Axiophot equipped with a Zeiss Axiocam digital camera) was used for photographing nickel-stained material as well as double exposures of ganglia with epifluorescence and differential interference contrast. Stacks of digital images of Neurobiotin™-stained wholemount tissues were collected with a confocal laser-scanning microscope (Leica TCS SP2) using ×10, ×20 multi-immersion, or ×40 oil-immersion objectives and the green line (543 nm) of the He/Ne laser. Stacks were merged using Leica Confocal Software™. The same software package was used to make 3D reconstructions and stereo images. Canvas™ (ACD Systems) was used to convert false-colour images to greyscale, to adjust brightness and contrast, to make line drawings, and to prepare the layout of all figures.

### Transmission electron microscopy

Tissues were fixed in 2.5% glutaraldehyde, 2.0% formaldehyde, and 0.025% CaCl_2_ in 100 mM cacodylate buffer (pH 7.2) for 2 h, postfixed for 1 h in 1% osmium tetroxide in phosphate buffered saline, washed in distilled water, stained for 1 h with 2% uranyl acetate in 70% ethanol, dehydrated in an ascending alcohol series, and transferred to propylene oxide (2 changes, 30 min. each). After incubation for 16 h in a 1:1 mixture of propylene oxide/epoxy resin (Epon, SERVA), and 2 changes of pure resin (2 h each) the resin was polymerised for 48 h at 57°C. Semi-thin sections (1 μm) and ultra-thin sections were cut on a Reichert OmU3 ultramicrotome. Semi-thin sections were counterstained in 0.5% methylene blue, 0.5% azur II, and 1% borax in distilled water. Ultrathin sections were collected on formvar-coated grids and treated with uranylacetate (2% in distilled water) for 20 min and lead citrate (0.2% in distilled water) for 7 min. Ultrathin sections were examined in a Zeiss EM10C microscope.

### Terminology

Naming of nerves follows the system of Campbell [13] that was later extended by Bräunig [5] and is further extended here. Muscles are named after Snodgrass [42]. The *superficial ventral inferior protocerebrum* (SVIP) was named by Ignell et al. [10].

## Competing interests

Both authors declare that they have no competing interests.

## Authors’ contributions

KK made the entire ultrastructural investigation and helped with the design of the figures. Everything else was done by PB. Both authors read and approved the final manuscript.
